# Impact of Breast Cancer Subtype Defined by Immunohistochemistry Hormone Receptor and HER2 Status on the Incidence of Immediate Postmastectomy Reconstruction

**DOI:** 10.1097/MD.0000000000002547

**Published:** 2016-01-22

**Authors:** Wei Wu, Shi Cheng, Heran Deng, Jiannan Wu, Kai Mao, Minghui Cao

**Affiliations:** From the Guangdong Provincial Key Laboratory of Malignant Tumor Epigenetics and Gene Regulation, Department of Breast Surgery, Breast Tumor Center (WW, HD, JW); Guangdong Provincial Key Laboratory of Malignant Tumor Epigenetics and Gene Regulation, Department of Anesthesiology (SC, MC); and Guangdong Provincial Key Laboratory of Malignant Tumor Epigenetics and Gene Regulation, Department of General Surgery, Sun Yat-sen Memorial Hospital, Sun Yat-sen University, Guangzhou, China (KM).

## Abstract

Immediate postmastectomy reconstruction has become an increasingly popular choice for breast cancer patients recently. However, whether molecular subtype of cancer impacts the incidence of breast reconstruction is unclear. We aimed to investigate the association between breast cancer subtype defined by immunohistochemistry hormone receptor (HR) and human epidermal growth factor receptor 2 (HER2) status and recent rates of immediate postmastectomy reconstruction in the United States.

The National Cancer Institute's Surveillance, Epidemiology, and End Results (SEER) database was used to evaluate stage I–III breast cancer patients with different subtypes who underwent either mastectomy alone or mastectomy plus reconstruction between 2010 and 2012. Univariate and multivariate analyses were conducted to identify factors influencing the incidence of immediate reconstruction.

Of 47,123 women included, 33.1% (10,712/32,376) of HR+/HER2−, 33.1% (1912/5768) of HR+/HER2+, 29.6% (850/2875) of HR−/HER2+, and 27.7% (1689/6104) of triple negative breast cancer patients received immediate breast reconstruction (chi-square test, *P* < 0.001), respectively. Thus, HER2-overexpressing and triple negative breast cancer patients received significantly less breast reconstruction. After adjusting for demographic, socioeconomic, geographic, or clinicopathologic factors, HER2-overexpressing (OR 0.896, 95% CI 0.817–0.984) and triple negative (OR 0.806, 95% CI 0.751–0.866) breast cancer patients remained less likely to undergo immediate postmastectomy reconstruction compared with HR+/HER2− or HR+/HER2+ patients. No significant difference was found in the type of reconstruction among different subtypes. Subgroup analysis showed that the difference of breast reconstruction rates among distinct subtypes varied with different grade and stage groups, and the association between breast cancer subtype and the reconstruction rate was not significant in low grade and early stage patients.

This population-based study determined that breast cancer subtype was an independent predictor for the utilization of immediate postmastectomy reconstruction. Patients with HER2-overexpressing or triple negative breast cancer subtype that has relatively higher risk of local recurrence, were less likely to receive immediate breast reconstruction than those with luminal tumors. Further studies are needed to disclose more underlying reasons of different reconstruction incidences for distinct subtypes of breast cancer.

## INTRODUCTION

Breast reconstruction reduces depression and improves quality of life in breast cancer patients.^1–3^ A number of reports showed similar rates of loco-regional recurrence (LRR), overall survival (OS), and disease-free survival (DFS) in patients treated with postmastectomy reconstruction or mastectomy alone.^4–6^ Some studies even identified that breast reconstruction was associated with significantly improved breast cancer-specific survival (BCSS).^7–9^ However, despite its established benefits, breast reconstruction rates across the United States vary from 15% to 42%.^10–13^ Although socioeconomic, geographic, and racial factors, as well as tumor stage and surgeon's characteristics associated with utilization of breast reconstruction have been extensively studied,^11–17^ the correlation between breast cancer subtype and immediate postmastectomy reconstruction rate remains unclear.

Breast cancer is a heterogeneous disease including several molecular subtypes, and distinct subtypes are correlated with significantly different outcomes and sensitivity to therapies.^18^ These molecular subtypes can be approximated by immunohistochemistry for estrogen receptor (ER), progesterone receptor (PR), and human epidermal growth factor receptor 2 (HER2) status.^19^ Although immediate breast reconstruction was found to improve well-being in breast cancer survivors without affecting the oncological safety of cancer treatment, concerns remain that it may delay adjuvant therapy and impair detection of local recurrence. A recent meta-analysis of 12,592 patients who underwent either breast-conserving surgery or mastectomy found that patients with HER2 positive or triple negative breast cancer had a higher risk of local recurrence than those with luminal subtype cancers.^20^ Furthermore, a single-institutional, retrospective study reported that breast cancer subtypes were independent prognostic factors for risk of local recurrence after immediate breast reconstruction, and it was suggested that the choice of postmastectomy reconstruction should be individualized according to breast cancer subtypes.^21^ Therefore, we hypothesized that HER2-overexpressing (ER/PR negative, HER2 positive) and triple negative breast cancer subtypes might be independent predictors for lower utilization of immediate breast reconstruction due to their higher risks of local recurrence compared with those of luminal subtypes. In this study, we analyzed a large national cohort of breast cancer patients using the Surveillance, Epidemiology, and End Results (SEER) database, to identify the recent trends in postmastectomy breast reconstruction among different subtypes across the United States.

## METHODS

### Patient Population

The SEER program of the National Cancer Institute provides cancer incidence, treatment, and survival data from population-based cancer registries covering ∼28% of the US population. The SEER 18 registry database (November 2014 submission) was used as the source of patient information for this study. Because the SEER database began collecting information on HER2 status since 2010,^22^ and the aim of this study was to evaluate the association between cancer subtype and recent incidences of immediate breast reconstruction, we limited cases diagnosed from 2010 to 2012. A total of 137,024 first primary invasive female breast cancer cases (International Classification of Diseases for Oncology, 3rd edition [ICD-O-3] histology codes 8000-8576, 8980-8981, and 9020/3) with American Joint Committee on Cancer (AJCC, 7th edition) stages I–III was identified. Patients with bilateral breast cancer, inflammatory breast cancer, unknown ER/PR status, unknown or borderline HER2 status, or unknown tumor or lymph node stage (T-/N-stage) were excluded. We also excluded patients diagnosed with breast cancer <18 years or >79 years, and patients who were not treated with mastectomy or mastectomy followed by immediate breast reconstruction (reconstruction within 4 months of mastectomy as defined by SEER). The remaining 47,123 patients formed the final study population.

A joint hormone receptor (HR) status using ER and PR statuses was created. Those with either ER or PR positive status (ER or PR positive groups included those with borderline results^23^ were grouped as HR positive, and those with ER and PR negative status were grouped as HR negative. We then classified all breast cancers into 4 subtypes: HR+/HER2−, HR+/HER2+, HR−/HER2+ (HER2-overexpressing), and HR−/HER2− (triple negative).^19^

Data collected comprises demographic and clinicopathological characteristics, treatment characteristics, and clinical outcomes. Data within the SEER were rendered anonymous, so the study was exempt from Sun Yat-sen Memorial Hospital Institutional Review Board review, and no informed consent was needed in this study.

### Statistical Analysis

Data were collected using the SEER∗Stat Software. We performed a descriptive analysis of the demographic and clinicopathological characteristics for the entire cohort of patients. The chi-square test was used as univariate analysis for identify potential factors influencing the incidence of immediate postmastectomy reconstruction. The multivariate logistic regression model was then used to define the breast cancer subtype's impact on the reconstruction rate by adjusting for other influential factors. The Akaike information criterion (AIC) and the bayesian information criterion (BIC) were calculated to select the best regression model. Subgroup analysis was performed to assess whether the difference of immediate breast reconstruction rate among distinct subtypes varied with different histologic grades and AJCC stages. The statistical analyses were conducted using the STATA 12.0 software (StataCrop, College Station, TX). All statistical tests were 2-sided, and statistical significance was defined as *P* < 0.05.

## RESULTS

Among the 47,123 patients treated with either mastectomy alone or mastectomy followed by immediate breast reconstruction between 2010 and 2012, 32,376 (68.7%) were HR+/HER2−, 5768 (12.2%) were HR+/HER2+, 2875 (6.1%) were HR−/HER2+ (HER2-overexpressing), and 6104 (13.0%) were HR−/HER2− (triple negative). A total of 15,163 (32.2%) patients received immediate postmastectomy reconstruction. The characteristics of the included patient population were summarized in Table 1. The median age was 56 (18–79) years, and most patients (89.3%) were from a metropolitan area. Totally, 58.8% of women had histologic grade I or II disease, and 78.2% had stage I or II breast cancer. Only 26% underwent postmastectomy radiation treatment.

**TABLE 1:**
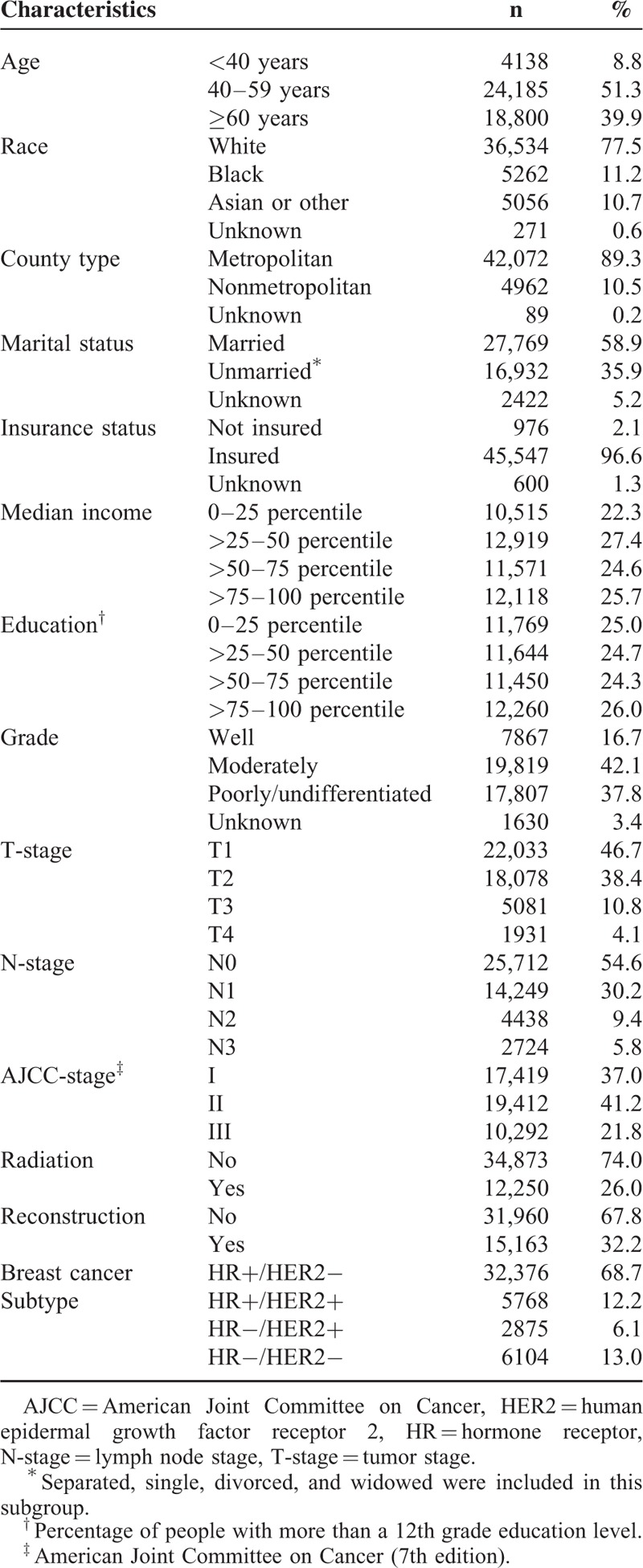
Characteristics of the Entire Study Population (n = 47,123)

**FIGURE 1:**
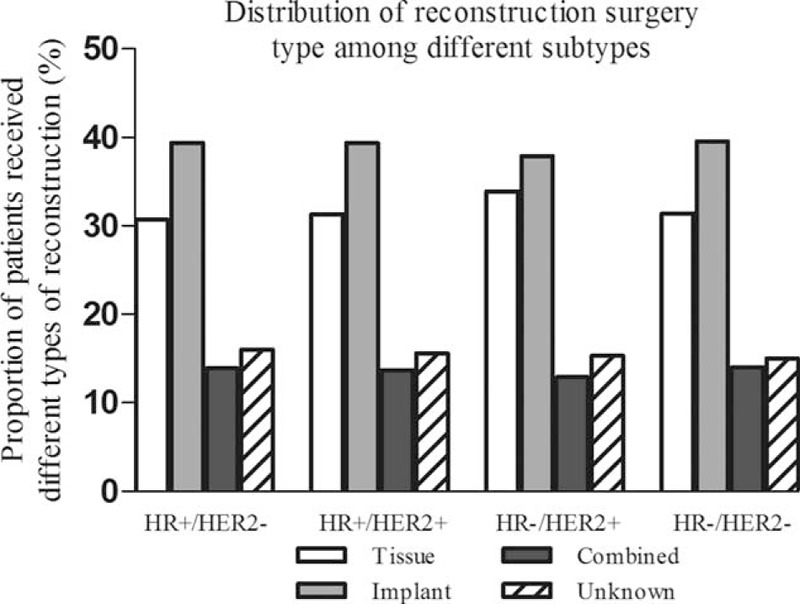
Distribution of immediate postmastectomy reconstruction type among different breast cancer subtypes.

**FIGURE 2:**
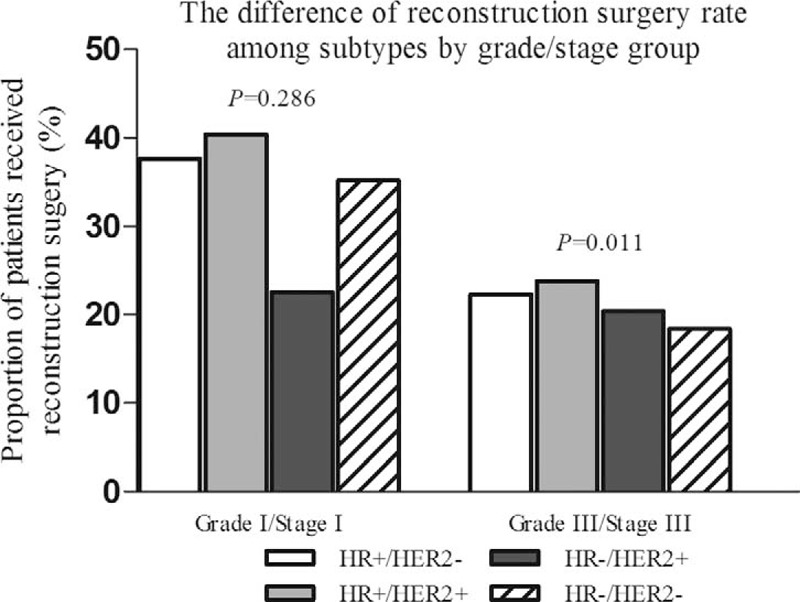
The difference of immediate breast reconstruction rates among breast cancer subtypes by distinct histologic grade and AJCC stage groups. AJCC = American Joint Committee on Cancer.

Of patients with HR+/HER2−, HR+/HER2+, HR−/HER2+ (HER2-overexpressing), and HR−/HER2− (triple negative) breast cancer, 33.1% (10,712/32,376), 33.1% (1912/5768), 29.6% (850/2875), and 27.7% (1689/6104) received immediate breast reconstruction surgery (chi-square test, *P* < 0.001), respectively. And the distribution of reconstruction surgery type among these 4 breast cancer subtypes was quite similar: the most common type was implant reconstruction, followed by tissue reconstruction, and the reconstruction combined by tissue and implant was the rarest (Figure 1). Univariate analysis showed that breast cancer subtype was significantly associated with utilization of immediate postmastectomy reconstruction (Table 2). Other factors found to be significant for the frequency of immediate breast reconstruction by univariate analysis were age, race, county type, marital status, insurance status, family income, education level, histologic grade, T-stage, N-stage, AJCC stage, and utilization of radiotherapy (*P* < 0.001 for all, Table 2). After adjusting for all these factors, we still identified that HER2-overexpressing and triple negative breast cancer patients were less likely to be treated with postmastectomy reconstruction compared with HR+/HER2− or HR+/HER2+ patients (HER2-overexpressing: OR (95% CI) 0.896 (0.817–0.984), *P* = 0.021; triple negative: OR (95% CI) 0.806 (0.751–0.866), *P* < 0.001, Table 2). Univariate and multivariate analyses defined no statistical difference of reconstruction rate between patients with HER2-overexpressing and triple negative breast cancer (chi-square test: *P* = 0.063; logistic regression: HER-overexpressing vs triple negative OR 1.106, 95% CI 0.995–1.230). Other predictors remained associated with higher immediate reconstruction rate by multivariate analysis were: diagnostic age< 40 years, white race, metropolitan area, married status, presence of health insurance, higher family income, higher education level, well or moderately histologic grade, earlier tumor or node stage, and lack of postmastectomy radiotherapy (*P* < 0.01 for all, Table 2). Subgroup analysis showed that the difference of breast reconstruction incidence among distinct subtypes varied with different histologic grade and AJCC stage groups (Figure 2). HER2-overexpressing and triple negative breast cancer women received less reconstruction surgery than luminal ones, in patients with high-grade tumor (poorly or undifferentiated) and advanced stage disease (AJCC stage III) (*P*=0.011), whereas there was no significant difference in reconstruction rates among the 4 subtypes in patients with low-grade tumor (well differentiated) and early stage disease (AJCC stage I) (*P* = 0.286, Figure 2).

**TABLE 2:**
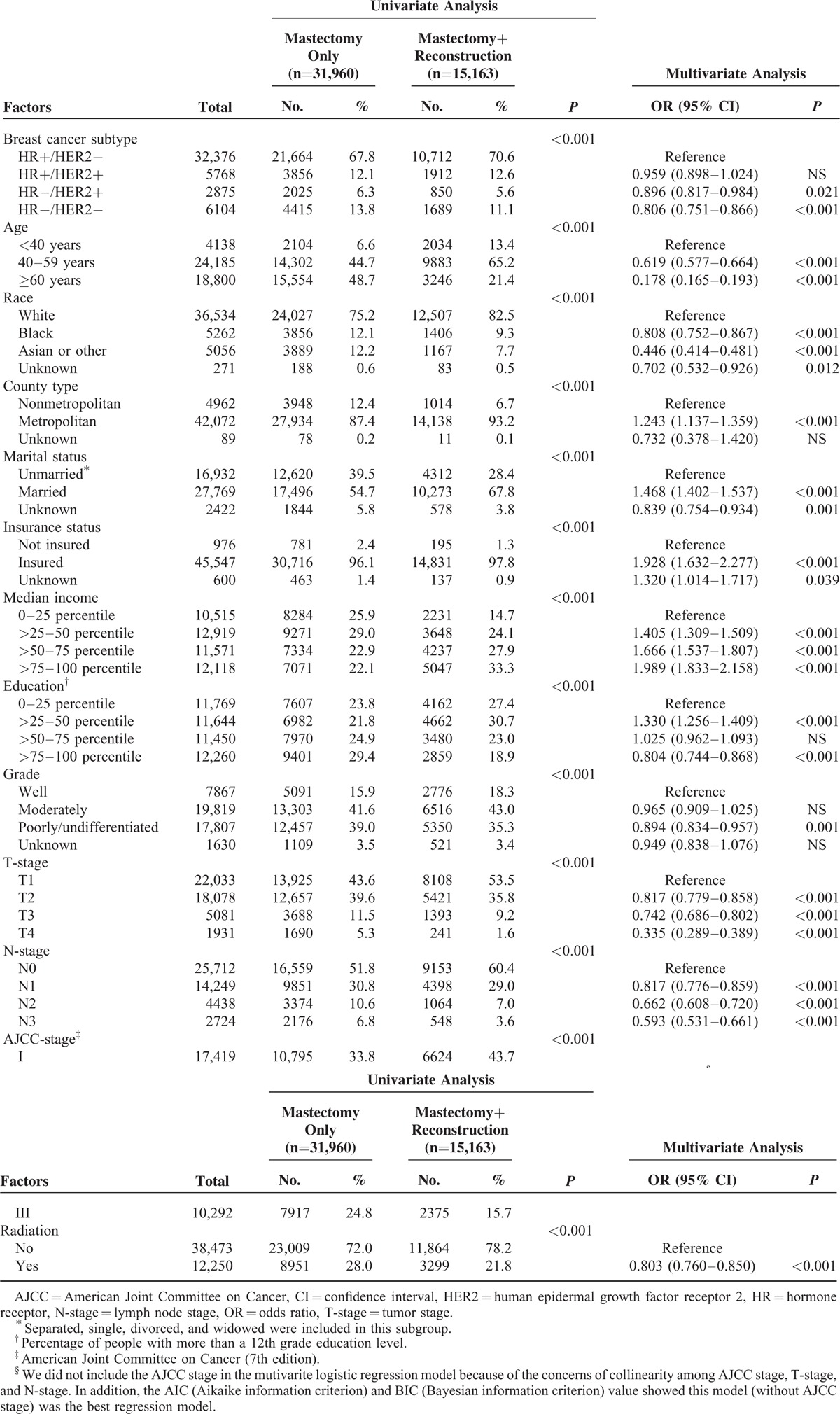
Factors Influencing the Utilization of Immediate Postmastectomy Breast Reconstruction

## DISCUSSION

To the best of our knowledge, the present study is the first population-based study to show that breast cancer subtype is an independent factor influencing the utilization of immediate postmastectomy reconstruction. Patients with HER2-overexpressing or triple negative breast cancer were less likely to receive immediate breast reconstruction surgery compared with those with luminal tumors. No significant difference was observed in the type of reconstruction among different subtypes in the modern practice. Moreover, our subgroup analysis demonstrated that the difference of immediate breast reconstruction rates among distinct subtypes varied with different tumor grade and disease stage, and the association between breast cancer subtype and reconstruction rate was not significant in low-grade and early stage patients.

Our results that socioeconomic, geographic, and racial factors, as well as diagnostic age, marital status, receiving of radiotherapy, and education level of patients are influential factors for the utilization of postmastectomy reconstruction is highly consistent with reports of previous population-based or multi-institutional studies.^11–14,16,17^ A new finding was the impact of breast cancer subtype defined by immunohistochemistry HR and HER2 status on the nationwide trends in immediate breast reconstruction. We found that HER2-overexpressing and triple negative breast cancer patients were significantly less likely to be treated with immediate postmastectomy reconstruction than those with luminal cancers. It is well-established that HER2-overexpressing or triple negative breast cancer developed more local recurrence disease and distant metastases than luminal subtypes.^20,24^ We and others^6,13^ also observed that high-risk patients with higher histologic grade or more advanced tumor or nodal disease received less immediate breast reconstruction treatment than low-risk women. This can be partially explained by the impact of local recurrence after reconstruction on the patients’ quality of life.^25^ Women might need to be treated with complicated reoperations or a new regimen of radiotherapy or systemic chemotherapy. Another possible reason is the patients’ concern about impairment of detection of local recurrence because of postmastectomy reconstruction. Morrow et al^16^ reported that nearly one-fourth of women who refused to undergo reconstruction after mastectomy because they feared about its potential interference with detection of recurrence despite the clinical evidence not supporting this contention.^26^ Furthermore, HER2-overexpressing and triple negative breast cancers are more likely to receive neoadjuvant chemotherapy because of their superiorities in systemic treatment response.^27,28^ Side effects or complications related to neoadjuvant chemotherapy certainly could impact decisions on immediate postmastectomy reconstruction. Unfortunately, data regarding the receipt of neoadjuvant chemotherapy was not available in SEER, so we cannot perform further in-depth analysis. However, our subgroup analysis identified that there was no statistical difference of reconstruction rate among breast cancer subtypes in patients with low-grade and early stage tumor, and this finding indirectly confirmed the above-mentioned potential reasons for the relatively lower trends of immediate postmastectomy reconstruction in triple negative or HER2-overexpressing patients.

Modern breast reconstruction modalities range in complexity and include prosthetics-based reconstruction (tissue expander and/or implant) and autologous tissue transfer reconstruction. A retrospective study using American College of Surgeons National Surgical Quality Improvement Program (ACS-NSQIP) database found that significant racial differences not only exist in the utilization of postmastectomy reconstruction, but also in the type of reconstruction.^17^ However, no significant difference was observed in the type of reconstruction among different subtypes in our study: the most common type was implant reconstruction, followed by tissue reconstruction, and the reconstruction combined by tissue and implant was the rarest. This might be due to the fact that the type of reconstruction relies more upon patient's body mass index (BMI) or surgical techniques rather than tumor characteristics.

Despite several strengths of this study including its population-based large sample size, novel insight into the impact of molecular subtype on the reconstruction rate, and more recent generalizable data (2010–2012), it had some limitations. First, the SEER database does not include margin status, neoadjuvant chemotherapy information, hospital name, or comorbidities such as obesity, diabetes, smoking status, as well as chronic cardiac or pulmonary disease, which may play a role in the utilization of breast reconstruction. Second, classification according to ER, PR, and HER2 status are only approximations of genotype-based molecular subtypes. Finally, several retrospective studies determined that physician bias may actually steer the patient toward the reconstructive option.^15,29,30^ Unfortunately, we cannot assess whether the surgeon's influence may affect the disparities of immediate reconstruction rate among breast cancer subtypes in this study.

In conclusion, the current population-based study demonstrated that in addition to the socioeconomic, geographic, racial, and tumor stage factors, breast cancer subtype was also an independent predictor for the utilization of immediate postmastectomy reconstruction. Women with HER2-overexpressing or triple negative breast cancer subtype that has relatively higher risk of local recurrence were less likely to receive breast reconstruction compared with HR+/HER2− or HR+/HER2+ patients. No significant disparity was observed in the type of reconstruction among different subtypes in the nationwide modern practice. Further studies are needed to disclose more underlying reasons for the different immediate reconstruction rates among distinct breast cancer subtypes.
